# Understanding PrEP Acceptability Among Priority Populations: Results from a Qualitative Study of Potential Users in Central Uganda

**DOI:** 10.1007/s10461-022-03606-8

**Published:** 2022-02-08

**Authors:** Radhika Sundararajan, Monique A. Wyatt, Timothy R. Muwonge, Emily E. Pisarski, Andrew Mujugira, Jessica E. Haberer, Norma C. Ware

**Affiliations:** 1 Center for Global Health, Weill Cornell Medicine, 402 East 67th Street, 2nd Floor, New York, NY 10065 USA; 2 Department of Emergency Medicine, Weill Cornell Medicine, New York, NY USA; 3 Department of Global Health and Social Medicine, Harvard Medical School, Boston, USA; 4 Harvard Global, Cambridge, USA; 5 Infectious Diseases Institute, Kampala, Uganda; 6 Department of Medicine, Massachusetts General Hospital, Boston, USA; 7 Department of Medicine, Brigham and Women’s Hospital, Boston, USA

**Keywords:** Pre-exposure prophylaxis, Uganda, HIV prevention, Priority populations, Scale-up

## Abstract

Daily oral pre-exposure prophylaxis (PrEP) can safely and effectively prevent HIV acquisition in HIV-negative individuals. However, uptake of PrEP has been suboptimal in sub-Saharan Africa. The goal of this qualitative study was to identify facilitators of and barriers to PrEP acceptability among target users not taking PrEP. Fifty-nine individuals belonging to Ugandan priority populations participated in a single in-depth interview. Participants perceived themselves as being at high risk for HIV acquisition, and expressed interest in PrEP as an HIV prevention strategy. Two forms of stigma emerged as potential barriers to PrEP use: (1) misidentification as living with HIV; and (2) disclosure of membership in a priority population. Acceptability of PrEP was dampened for this sample of potential PrEP users due to anticipated stigmatization. Mitigating stigma should be a key component of effective PrEP delivery to reach UNAIDS goal of ending the AIDS epidemic by 2030.

## Introduction

Daily oral pre-exposure prophylaxis (PrEP) can safely and effectively prevent HIV acquisition in HIV-negative individuals [1–4]. PrEP implementation programs have demonstrated the feasibility of delivering PrEP in a variety of settings [5–8]. If implemented successfully, use of oral PrEP among Ugandan female sex workers (FSW), serodiscordant couples (SDC), and adolescent girls and young women (AGYW), alone, could prevent nearly 9% of anticipated HIV infections from 2018 to 2030 [9]. While the number of countries implementing PrEP has increased in recent years, sub-Saharan Africa accounts for only 44% of global PrEP initiations despite accounting for more than 70% of the global burden of HIV infection [10, 11]. PrEP uptake, and especially persistent use of PrEP over time, has been suboptimal in sub-Saharan Africa [12], despite wide-ranging delivery strategies that have increasingly targeted a variety of priority population groups. These include AGYW [13], HIV-negative partners in SDC [5], pregnant women [14], FSW, and men who report having sex with men (MSM) [7].

Prior research in sub-Saharan Africa has traced expressed interest in PrEP to distinct facilitators that vary across populations. PrEP demand among HIV SDC has been driven by a desire to preserve trust and intimacy within partnered relationships [5, 15–17], and facilitate conception without risk of HIV transmission [18–21]. Individuals involved in sex work have shown interest in PrEP to mitigate occupational risks of HIV acquisition [22–25]. Among some AGYW [26], PrEP is considered a discreet modality for HIV prevention, compared to condoms [27]. Among MSM, demand for PrEP stems from the desire for an additional ‘layer of protection’ against HIV for themselves and their partners [28, 29]. Previous studies have also identified barriers to PrEP use, including geographic distance from clinics, concerns about side effects, long queues at clinics, discrimination from health center staff, and cost of PrEP [30–32].

To effectively deliver PrEP at scale, we must understand why implementation of PrEP is currently falling short of target levels. This qualitative study examined PrEP acceptability among potential PrEP users in higher risk, priority populations in Uganda. We explore perceptions of PrEP in specific priority population subgroups for the purpose of characterizing facilitators of and barriers to PrEP use in order to inform PrEP scale-up early in the rollout process.

## Methods

### Study Design and Setting

In Uganda, PrEP was rolled out nationally to HIV-negative partners in serodiscordant couples in 2017 [5]; it has since been made available to other high-risk, priority population groups. The Barriers Study was designed to assess knowledge, acceptability and potential facilitators of and barriers to PrEP uptake and adherence among potential users who were eligible for, but not taking, PrEP. The Barriers Study was implemented by Makerere University’s Infectious Diseases Institute Kasangati research site (IDI-Kasangati), which is located near Kampala, in central Uganda [33].

**Fig. 1:**
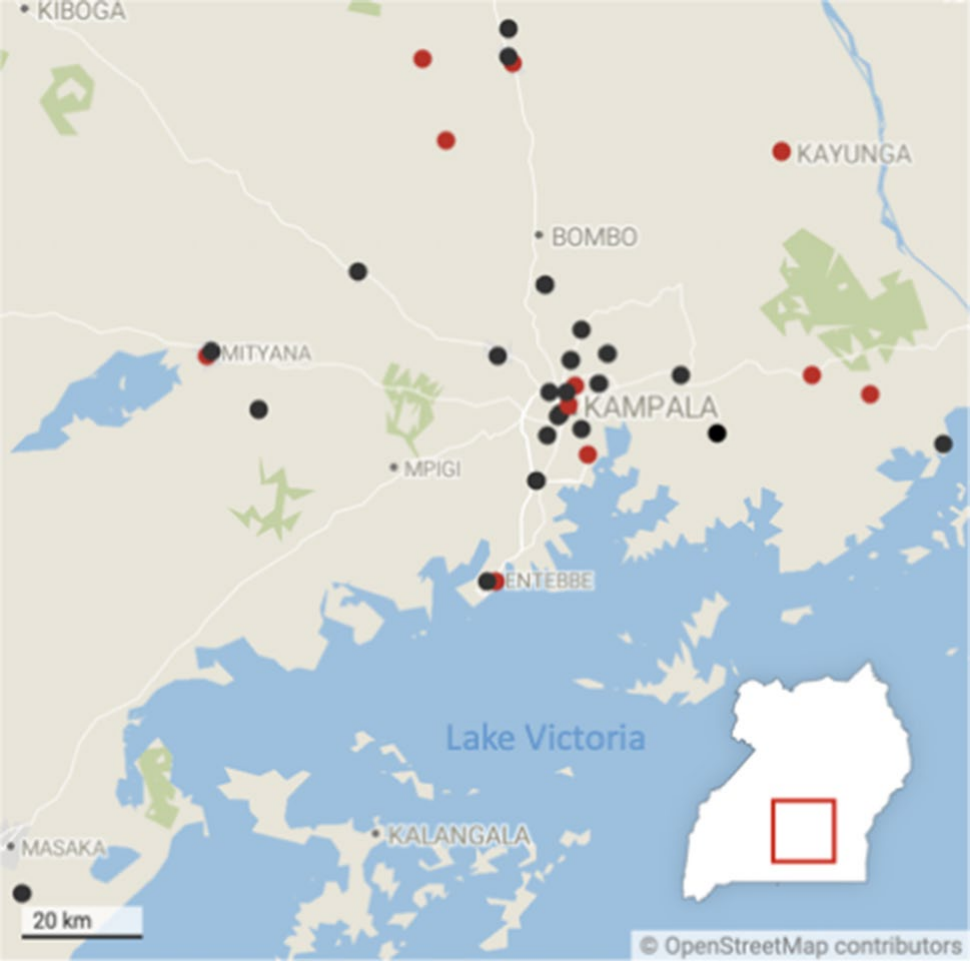
Map of study recruitment areas in Central Uganda. Dots indicate hospitals (red) and health centers (black) where PrEP is distributed (Color figure online)

The recruitment catchment area for the Barriers Study consisted of 35 health centers selected from facilities that were providing HIV services but not yet offering PrEP, and were located within a 70-km radius of the IDI-Kasangati study site. The centers were stratified in roughly equal proportions by urban, peri-urban, and rural locations. The Barriers Study included 250 study participants from four priority populations: (1) HIV-negative partners in serodiscordant couples (SDC) (N = 53); (2) female sex workers (FSW) (N = 56); (3) men who report having sex with men (MSM) (N = 75), and (4) fisherfolk (FF) ﻿(N = 66). When the study was conducted, only these four priority populations were eligible for PrEP per Ugandan Ministry of Health guidelines [34]. Participants were aged 18 and older and were recruited from communities surrounding these 35 planned PrEP delivery sites (Fig. 1) [33].

The research reported here represents the qualitative component of the Barriers Study. The goal of the qualitative component was to characterize facilitators of and barriers to PrEP acceptability among potential users as PrEP was being rolled out in Uganda. These data also provide contextual information to explain quantitative findings from the Barriers study [33].

### Qualitative Sampling and Recruitment

Using purposeful sampling [35], we identified a subsample of Barriers Study participants to take part in the qualitative study. Participants who reported having sexual intercourse at least once in the previous month, and correctly identified PrEP as a pill to prevent HIV infection in the Barriers Study baseline questionnaire were included in the qualitative sampling scheme. Individuals were then selected to systematically represent the four priority groups included in the Barriers Study. Research assistants (RAs) contacted prospective qualitative study participants by telephone, inviting them to participate. Sixty participants were recruited into the qualitative study, with 15 individuals representing each group of prospective PrEP users. One participant from the SDC group was excluded after disclosing that she was living with HIV, and therefore not a potential PrEP user. Sample size was guided by research indicating that thematic saturation can be achieved with completion of 12–15 qualitative interviews within a group [36].

### Data Collection

Data collection consisted of a single in-depth qualitative interview. All interviews were conducted in-person by trained Ugandan RAs fluent in the local language (Luganda) and took place in private locations of participants’ choosing to maintain privacy and confidentiality. An interview guide was used to guide the conversation, and consisted of open-ended questions and probes to encourage participants to elaborate on responses. Interviews lasted approximately one hour, and explored the following topics: (1) knowledge and attitudes about PrEP; (2) perspectives on PrEP uptake and adherence; (3) sexual behavior and HIV risk; and (4) HIV prevention strategies. Interviews were audio-recorded, translated and transcribed directly from Luganda into English by the interviewer. Qualitative interviewees were assigned a unique study ID, and no personal identifying information was included in the transcript data. Interview transcripts were reviewed by MAW and EEP  to monitor data quality and ensure that interview content was aligned with qualitative study goals. RAs participated in weekly supervision calls and received regular feedback on interviewing and transcription technique. Data collection took place from November 2017 through August 2018. Fifty-nine interviews were completed.

### Data Analysis

We used an inductive, content analytic approach for data analysis [37]. All interview transcripts were reviewed to develop a coding scheme. Data were coded in Dedoose [38] by a team of Ugandan RAs, overseen by EEP. One quarter of transcripts were coded by two members of the research team and the results compared to ensure consistent use of the coding scheme. Discrepancies were resolved through discussion. A framework approach [39] was also used to organize coded data by interview topic. Coded data were entered into a working analytical matrix by the first author, RS. The first and second authors (RS and MAW) reviewed the matrix independently to identify emerging concepts, which were then grouped inductively to develop categories that informed PrEP acceptability among potential users.

### Ethics Approval

This study was reviewed and approved by the Massachusetts General Hospital/Partners Human Research Committee (#2017/P000482/PHS) (Boston, USA), National HIV/AIDS Research Committee (#ARC196) (Kampala, Uganda) and the Uganda National Council for Science and Technology (#SS 4277) (Kampala, Uganda). All participants provided written informed consent for the Barriers Study at enrollment; separate written consent was obtained for the qualitative interview immediately before the interview took place.

## Results

### Participant Characteristics

Participant characteristics are shown in Table 1. Our sample of 59 potential PrEP users were mostly male (68%), between 26 and 40 years of age (61%), unmarried (51%), and recruited from urban regions (51%). More SDC participants were married, recruited from peri-urban or rural areas, and older than participants from other groups. Otherwise, characteristics across subgroups were similar. The majority of participants perceived themselves to have moderate or high risk of HIV acquisition over their lifetimes (76%), and in the coming year (66%). Nearly all had previously tested for HIV (98%).

**Table 1:** Characteristics of study participants

Characteristics	Type of participant (N = 59)				
	MSM (N = 15)	FSW (N = 15)	FF (N = 15)	SDC (N = 14)	Overall (N = 59)
*Enrollment site*					
Urban	8 (53%)	11 (73%)	7 (46%)	4 (29%)	30 (51%)
Peri-urban	3 (20%)	3 (20%)	4 (27%)	7 (50%)	17 (29%)
Rural	4 (27%)	1 (7%)	4 (27%)	3 (21%)	12 (20%)
*Female gender*	0 (0%)	15 (100%)	4 (27%)	5 (36%)	24 (41%)
*Age group (in years)*					
< 25	6 (40%)	2 (14%)	5 (33%)	2 (14%)	15 (25%)
26–40	8 (53%)	11 (73%)	9 (60%)	8 (57%)	36 (61%)
40 >	1 (7%)	2 (13%)	1 (7%)	4 (29%)	8 (14%)
*Marital status*					
Single	11 (73%)	12 (80%)	5 (34%)	2 (14%)	30 (51%)
Married	4 (27%)	1 (7%)	8 (53%)	12 (86%)	25 (42%)
Separated	0 (0%)	2 (13%)	2 (13%)	0 (0%)	4 (7%)
*Perceived lifetime risk of HIV acquisition*					
High	10 (67%)	12 (80%)	7 (47%)	9 (64%)	38 (64%)
Moderate	1 (7%)	0 (0%)	4 (26%)	2 (14%)	7 (12%)
Low	4 (26%)	3 (20%)	3 (20%)	2 (14%)	12 (20%)
No risk	0 (0%)	0 (0%)	0 (0%)	0 (0%)	0 (0%)
Don’t know	0 (0%)	0 (0%)	1 (7%)	1 (8%)	2 (3%)
*Anticipated HIV acquisition risk (in next 1 year)*					
High	4 (27%)	7 (47%)	7 (47%)	5 (36%)	23 (39%)
Moderate	4 (27%)	3 (20%)	5 (33%)	4 (29%)	16 (27%)
Low	6 (40%)	4 (27%)	2 (14%)	3 (21%)	15 (25%)
No risk	1 (6%)	1 (6%)	0 (0%)	0 (0%)	2 (4%)
Don’t know	0 (0%)	0 (0%)	1 (6%)	2 (14%)	3 (5%)
*Ever tested for HIV*					
Yes	15 (100%)	15 (100%)	14 (93%)	14 (100%)	58 (98%)
No	0 (0%)	0 (0%)	1 (7%)	0 (0%)	1 (2%)

Percentages indicate proportional distribution within columns

### Overview of Results

**Fig. 2:**
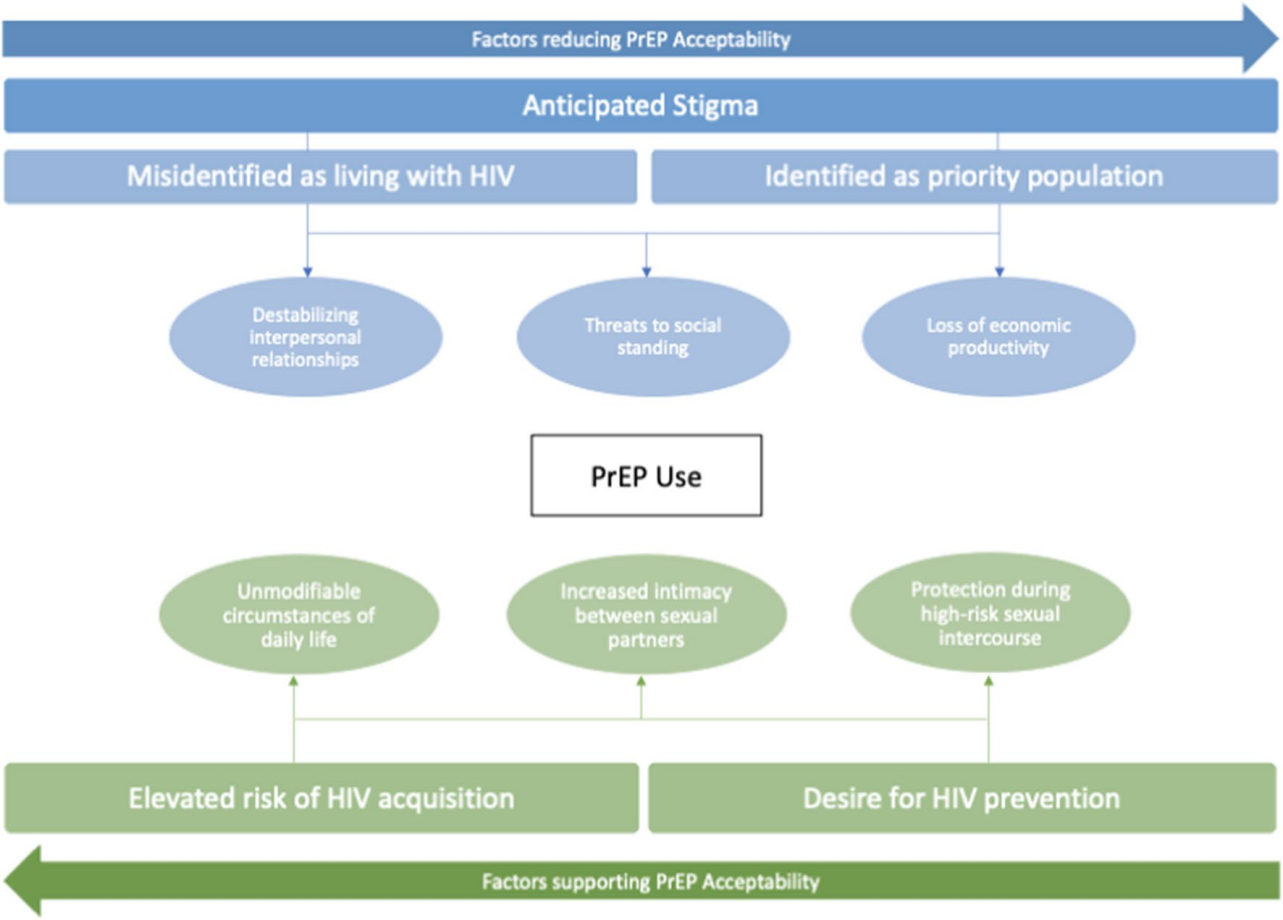
Diagram of findings relating to acceptability of PrEP

Our analysis identified facilitators of and barriers to PrEP uptake, shown in Fig. 2, which were cross cutting across potential users. A perception of being at high risk of HIV acquisition, and resulting high interest in PrEP as HIV prevention facilitated acceptability. At the same time, worry about being misidentified as living with HIV and/or identified as a member of a higher risk priority population presented obstacles to acceptability. Anticipated stigma emerges from this analysis as a major barrier to PrEP uptake and use. These results are presented in greater detail below.

### Facilitators of PrEP Use

#### Prospective PrEP Users Perceived Themselves to be at High Risk for HIV Acquisition

In this sample of prospective PrEP users, individuals generally felt at elevated risk for HIV acquisition. Being at high risk for HIV acquisition was sometimes described as an unmodifiable circumstance. Participants perceived that HIV risk was inherently linked to their lived experiences and in many cases was unavoidable. For example, condomless intercourse was expected within partnered relationships. HIV-negative partners of SDC felt particularly vulnerable to HIV acquisition as a result. One woman stated,

I have sex with an infected man, and most of the time he refuses to use condoms … I live with an infected partner. Anytime I can get HIV because I do not use anything to protect myself from HIV. (female, aged 38, SDC group)

Among some MSM participants, condomless intercourse and multiple sexual partners were described as common practice within the gay community. Therefore, self-identifying as MSM was perceived as linked to having increased risk of HIV acquisition:

[I felt most at risk] when I joined the gay community. I was so excited, moving here and there, parties, outings, dates. I remember I slept with three people in 1 week. One day I wondered whether I had contracted HIV… I thought a lot about it. I used a condom with only one of the three people I slept with. (male, aged 23, MSM group)

Perceived HIV risk was also heightened when risk was considered outside one’s control—e.g., when there was mistrust within a relationship, or when the HIV status or sexual activities of partners were unknown. This was evidenced in the following excerpts from participants in the FSW and MSM groups:

I am at risk of getting HIV because not everybody that I have sex with, his HIV status is not known to me since I do not have HIV testing kits with me. (female, aged 24, FSW group)

I think about my partners. You may trust them, but they may involve themselves with one person that’s infected. That’s why I need PrEP. (male, aged 23, MSM group)

#### Interest in PrEP was High

Overall, prospective users in this sample reported high interest in PrEP as HIV prevention because they felt at substantial risk of acquiring HIV. PrEP was appealing because it provided a sense of protection during condomless intercourse, or when a partner’s HIV status was unknown. PrEP was described as a weapon to defend oneself proactively against HIV acquisition, rather than waiting to become infected:

You cannot be with a snake in the house and have nothing to protect you. It can bite you. But when you have this small stick, you can hit it anytime and stop it from biting you …. Someone gives you the stick to protect yourself because the snake can bite you at any time. The snake is the HIV and the stick that I want is PrEP. (male, aged 31, SDC group)

PrEP was also described as an alternate form of “security” during sex, e.g., when condoms broke during intercourse or when condom use could not be safely negotiated due to the threat of interpersonal violence:

A client takes you … And they do not want to use condoms. You do not know their [HIV] status and there is no way you can leave, and you just have to give into the live [unprotected] sex to avoid being killed or assaulted … PrEP is good because it helps you prevent before the exposure. Wherever you go, you know that at least you have security in your body. (female, aged 33, FSW group)

For other prospective users, PrEP was desired as a means of facilitating unprotected sex between partners, while protecting against HIV acquisition. Condoms were perceived to interfere with sexual pleasure and intimacy, and attempts at procreation. In partnered relationships, PrEP could support intimacy by fostering physical and sexual connections between couples:

Condoms break intimacy, yet PrEP promotes intimacy during sex because you have it live, to enjoy and get satisfied that you have been with someone you love .... With PrEP you stay natural. You have sex as it is supposed to be, unlike using condoms where you do not feel each other. (male, aged 43, FF group)

### Barriers to PrEP Use

#### PrEP was Unfamiliar and Mistaken for HIV Treatment

Despite perceived need and interest in PrEP for HIV prevention, potential users acknowledged obstacles to acceptability. Taking oral medicine for HIV prevention was an unfamiliar concept to most people within their communities. Large, colored tablets were readily identified as antiretroviral treatment (ART) for HIV. Across priority population groups, prospective users were concerned that others would be unable to differentiate between PrEP and ART, resulting in misidentification as a person living with HIV. This could be damaging in a number of ways. For example, being seen taking daily medications could result in being labeled as a “sick person”—i.e., living with HIV:

They would say “You see that one, she is on tablets”. Sometimes you can accidentally cause harm to him or her, and because this person saw you taking medicine, will immediately tell you that you are infected. It will be an abuse. It hurts for people to think that I am infected, yet I am not. (female, aged 46, FSW group)

Further, being misidentified as living with HIV was described as a potential threat to one’s reputation by “creating a bad name” within one’s community, leading to social isolation and/or feelings of shame. FSW feared that being labeled as living with HIV would “scare” away customers, resulting in economic hardship. For example, one FSW stated,

Because PrEP is ARVs, it can even scare our customers away when they see us taking it because they think we are infected [with HIV]. (female, aged 24)

Many participants were particularly concerned that use of PrEP would jeopardize interpersonal relationships. Mistaking PrEP for HIV treatment was anticipated to sow discord between intimate partners, potentially leading to separation. One fisherfolk man stated:

My wife cannot see [me taking PrEP] because she may think I have HIV and I am taking ARV for treatment even if I tell her it is for prevention. (male, aged 43, FF group)

It could also cause unnecessary worry among family members, who may believe PrEP users are terminally ill and facing imminent death:

People are not familiar with PrEP … people always attach meaning to something that is new to them. I may go to the village and they check in my bag and once they see that tin of PrEP they would say “this one is infected” .... It is hard if they get to know because they will be worried that their son is dying soon. Yet, I do not want them to get worried and in that way, I may fail to take PrEP. (male, aged 23, SDC group)

#### HIV Prevention (PrEP) was Intended for Higher Risk, Priority Populations

Prospective users were aware that PrEP was only available to populations with elevated HIV risk, and not accessible to everyone. Taking PrEP would therefore signal association with a priority population. This was feared to lead to disclosure of behaviors or identities that might invite feelings of shame, or worse, trigger abuse or discrimination by others.

I know there are many who fear being seen with PrEP because people will think …. they belong to our [MSM] community and discriminate against them. I am telling you, there are many people who are homophobic. (male, aged 27, MSM group)

More specifically, these concerns were prominent among MSM and FSW potential users, who worried that taking PrEP for HIV prevention would label them as “Kuchu” (slang for gay or lesbian) or a sex worker. Preferring to keep their sexual identities or behaviors “silent,” potential users indicated they would ultimately decline PrEP so as to avoid this negative association.

I want to keep my sexual status silent. I would not want people to know I am kuchu .... if I am taking PrEP and I disclose to people that it is for HIV prevention, they may know that I am a kuchu, which I do not want. (male, aged 27, MSM group)

Many people do not know PrEP, and that is why I asked the last time you were here whether PrEP is for us sex workers alone or it’s even for other usual people. I would be scared taking medicine that is unique to people, because not so many people would like to associate themselves with sex work or with sex workers. (female, aged 38, FSW group)

In limiting access to PrEP, HIV prevention programs signal that PrEP is not intended to be used by “usual” or “ordinary” people. PrEP is seen as a marker of difference. As one woman in the FSW group observed,

PrEP is intended to be used by key populations, but even other people, the ordinary people we see or that stay home – the married people – would also be helped by PrEP. Personally, I see married women as at risk because their husbands sleep around… We are all at risk of HIV. (female, aged 31, FSW group)

Participants in this study suggested that PrEP would be more acceptable to them if it were made available to any HIV-negative, sexually active individual at risk, rather than only specific populations:

What health workers need to do is to sensitize people about PrEP so that people get to know the truth that PrEP is for prevention, not for HIV treatment, and that a person who picks it from the health centre is HIV negative and is preventing against HIV ... I would wish PrEP to be [available] for everyone engaging in sexual acts. (male, aged 30, FF group)

These stigmatized identities were described as acting in overlapping ways to potentially undermine an individual’s relationships, social standing, and economic productivity. Participants describe how dual forms of stigma make the potential user vulnerable to discrimination and isolation, creating significant barriers to PrEP use:

I do not want my family members to know the kind of work I do. It is shameful if my children get to know that I am doing sex work … There may be someone who knows that PrEP is for prostitutes. That person will spread to the rest, “I saw so-and-so taking PrEP, so she is a prostitute” … Because PrEP is ART, it can even scare our customers when they see us taking it because they will think we are [HIV] infected. (female, aged 24, FSW group)

## Discussion

The goal of this study was to elucidate facilitators of and barriers to PrEP use among potential users. We examined a range of perspectives among 59 members of four priority populations who were eligible, but not using, PrEP in central Uganda, early in the PrEP rollout process. Potential users perceived themselves at high risk of acquiring HIV and were interested in using PrEP for HIV prevention. However, anticipated stigma emerged as a major obstacle to PrEP use for all groups despite perceived HIV risk and interest in HIV prevention. We identified two forms of anticipated stigma relevant to PrEP use. First, willingness to take PrEP as an HIV prevention strategy was dampened by fears of being misidentified as living with HIV. Second, use of PrEP was feared to disclose membership in a priority population. Both of these stigmatized identities were perceived to threaten interpersonal relationships, social standing, and economic productivity. Together, these two forms of stigma could tip the balance, discouraging use of PrEP when anticipated threats outweigh perceived benefits.

Anticipated stigma was reported as the predominant barrier to PrEP acceptability among study participants. This is notable considering the diverse membership of the four priority populations (sex workers, MSM, fisherfolk and HIV-negative serodiscordant partners). All feared that being misidentified as living with HIV would result in isolation or discrimination. Our data align with prior work illustrating how PrEP use is perceived as “marking” the user in stigmatizing ways [40–44]. These findings underscore significant levels of HIV-related stigma that still exist within these marginalized communities in Uganda [42]. Furthermore, PrEP in Uganda is identical to ART for HIV treatment (tenofovir/lamivudine), such that PrEP users are indistinguishable from people living with HIV. Fears of being misidentified as living with HIV have been reported among PrEP users in many other parts of sub-Saharan Africa [23, 44–49]. Our data illustrate that despite perceived need for PrEP as HIV prevention, anticipated stigma can interfere with PrEP uptake use among target users. Similarly, stigma can also undermine PrEP adherence and motivate discontinuation [48], thereby hindering the effectiveness of PrEP as HIV prevention.

Our data also point to another aspect of anticipated stigma, in which PrEP use implied membership in a priority population. Individuals in MSM and FSW participant groups were hesitant to use PrEP because it could lead to unintended disclosure of sexual orientation or involvement in sex work. This concern was less prominent among FF participants or partners in SDCs, though all groups acknowledged PrEP had an undesired association with higher risk, priority populations. In other settings, PrEP users have described a stigma associated with “immoral” sexual behaviors presumed among priority population [50, 51]. PrEP as a marker of priority population membership is especially threatening for MSM and FSW because same-sex relations and sex work are criminalized in Uganda [52], further complicating PrEP delivery as a result of fear of prosecution or incarceration.

Our results can be framed using the Healthcare Stigma and Discrimination Framework [41], a theoretical model positing a stigmatization process that cuts across health conditions. The process traces the impact of negative “drivers” as the “marking” of individuals in a way that results in stigma being “manifested” as particular experiences or practices (e.g., exclusion from a social group). These experiences and/or practices negatively affect desirable outcomes, such as access to health care or other health-enhancing resources. Our data suggest that drivers of stigma for potential PrEP users include fear of social isolation, economic hardship, community lack of knowledge about PrEP, and social judgement. Individuals are “marked” by intersecting stigma, i.e., potential mis-identification as living with HIV *and* disclosure as a member of a priority population. These stigma experiences are manifested as anticipated stigma with the outcome being unwillingness to use PrEP. Application of this framework can guide development of stigma-reduction interventions focused on reducing drivers of stigma in order to improve health outcomes.

Currently, many PrEP rollout programs in Africa are targeted to higher risk, priority populations, inherently linking PrEP use to stigmatized identities. We have shown that these negative associations are a significant barrier to PrEP use among potential users. Similarly, policies emphasizing HIV risk as a determinant for PrEP have been criticized as undermining implementation, resulting in critical gaps in coverage [53–57]. Amico and Bekker, for example, described global PrEP rollout as suffering from “flawed messaging” [56]. Despite this, programs can be modified to minimize stigma. For example, presenting PrEP as a lifestyle choice instead of a biomedical tool may improve uptake among AGYW [58]. In South Africa, a PrEP campaign was designed with affirming messages about personal agency, rather than HIV risk, effectively stimulating interest in PrEP among AGYW [59]. PrEP messaging in Kenya has shifted away from mitigation of HIV risk to emphasize empowerment and health preservation; PrEP eligibility is currently guided by “self-selection” for elevated risk, eliminating the criterion of membership in a key population [60]. We found that potential users were interested in using PrEP for HIV prevention and acknowledged their increased risk of acquisition. Potential users in our study also endorsed normalizing PrEP messaging, suggesting it may be effective in increasing PrEP use in Uganda.

We investigated facilitators of and barriers to PrEP acceptability among a diverse group of potential users in Uganda, describing a perceived need for PrEP as HIV prevention, while illustrating two forms of anticipated stigma which mitigate acceptability. We acknowledge the following study limitations. Data were collected before PrEP services were widely available in Uganda, and therefore reflect knowledge and experiences among potential users prior to national rollout. However, recent studies in eastern Africa demonstrate that PrEP continues to be mistaken for HIV treatment rather than prevention [40–42]. Additionally, since PrEP is currently only available to priority populations in Uganda [61], anticipated stigma will likely persist as a barrier to PrEP uptake, underscoring that the findings from this qualitative analysis are directly applicable to current program implementation. Further work is needed to examine the impact of stigma in healthcare facilities on the uptake of PrEP, and the role of targeted stigma-reduction efforts involving healthcare providers. Finally, this work was conducted among potential PrEP users in a region of central Uganda and may not be generalizable to other contexts. However, we noted similar experiences of stigma among PrEP users throughout sub-Saharan Africa [45–47, 50, 51].

## Conclusions

Potential PrEP users in Uganda perceive a need for PrEP as HIV prevention. Willingness to take PrEP is dampened by two forms of anticipated stigma: (1) misidentification as living with HIV; and (2) disclosure of membership in a priority population. Stigma may shift the balance away from using PrEP, as potential users could perceive these threats to outweigh the benefits of the medication. Effective PrEP delivery and implementation will require stigma reduction, which may be achieved through expanding PrEP eligibility beyond priority populations, and health campaigns emphasizing PrEP as part of maintaining health. By reducing stigma surrounding PrEP, implementation programs may have more success reaching the UNAIDS goal of ending the AIDS epidemic by 2030.

## Data Availability

Deidentified data are available upon reasonable request to the corresponding author.
